# P-1656. Different Drugs for Different Bugs? A Comparison of a Solid Organ Transplant Population-specific Antibiogram with the Hospital-wide Antibiogram

**DOI:** 10.1093/ofid/ofae631.1822

**Published:** 2025-01-29

**Authors:** Scott Borgetti, Arzina Aziz Ali, Alan E Gross, Alfredo J Mena Lora, Ryan D Knodle, Nahed Ismail, Taha Ali

**Affiliations:** University of Illinois at Chicago, Chicago, Illinois; University of Illinois at Chicago, Chicago, Illinois; University of Illinois, Chicago, IL; University of Illinois Chicago, Chicago, Illinois; University of Illinois at Chicago, Chicago, Illinois; University of Illinois at Chicago, Chicago, Illinois; Northwest Suburban Medical Associates, Chicago, Illinois

## Abstract

**Background:**

Solid organ transplant (SOT) recipients are vulnerable to infections with multi-drug resistant organisms. Studies have shown that SOT patients often do not receive adequate empiric antimicrobials for serious Gram-negative infections. Knowledge of epidemiologic differences in resistance rates in this population may help guide empiric antibiotic choices for SOT patients.

Cohort A

**Figure:**
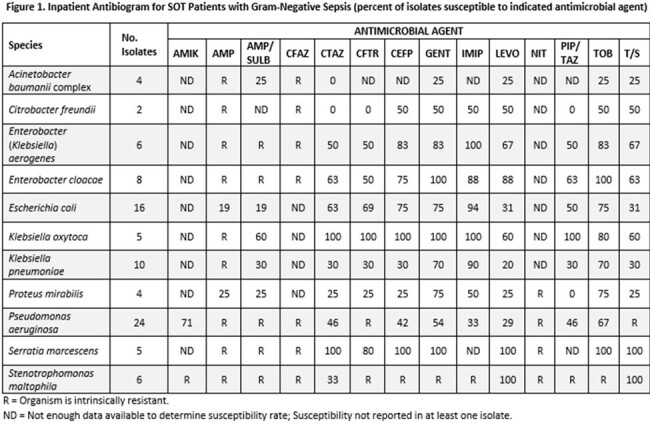

**Methods:**

We performed a retrospective cohort study comparing antibiotic susceptibility rates of Gram-negative sepsis in SOT patients (cohort A) between 1/1/2021 and 4/30/2023 to the hospital-wide inpatient antibiogram for 2021-2022 (Cohort B). Using resistance rates from Cohort A, we developed a weighted-incidence syndromic combination antibiogram (WISCA) comparing SOT population specific empiric antibiotic therapy to the institutional empiric antibiotic recommendations for non-SOT patients with sepsis (figure 3).

Cohort B

**Figure:**
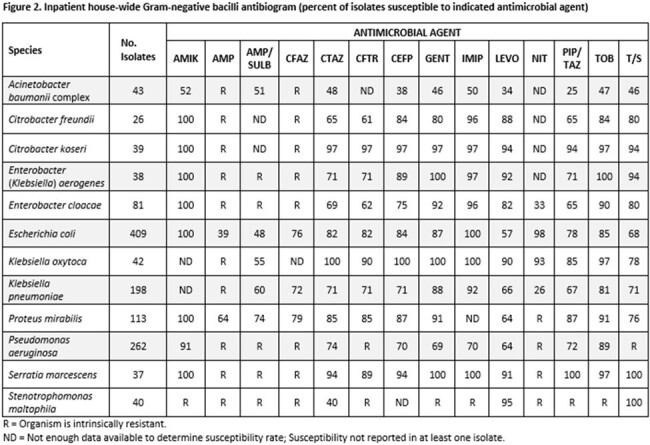

**Results:**

Cohort A had 90 Gram-negative isolates compared to 1328 in cohort B. Pseudomonas (27%) and E.coli (31%) were the most common isolates in cohorts A and B respectively. Pseudomonas isolates in cohort A were less likely to be susceptible to common anti-pseudomonal beta-lactams compared to cohort B, including piperacillin-tazobactam (46% vs 72% susceptible; OR 0.33, 95%CI 0.13-0.83), imipenem (33% vs 70% susceptible; OR 0.21, 95%CI 0.08-0.56), and cefepime (42% vs 70% susceptible; OR 0.31, 95%CI 0.12-0.80). E. coli isolates in cohort A were less likely to be piperacillin-tazobactam (50% vs 78% susceptible; OR 0.28, 95%CI 0.09-0.89) susceptible than isolates in cohort B, but not significantly less likely to be susceptible to imipenem (94% vs 100% susceptible; OR 0, 95%CI 0-1.53) or cefepime (75% vs 84% susceptible; OR 0.57, 95%CI 0.17-2.5). WISCA analysis of Gram-negative sepsis for SOT patients yielded imipenem (62% of isolates covered) as the optimal beta-lactam as opposed to the institutional recommendation of piperacillin-tazobactam (only covered 39% of SOT isolates).

**Figure:**
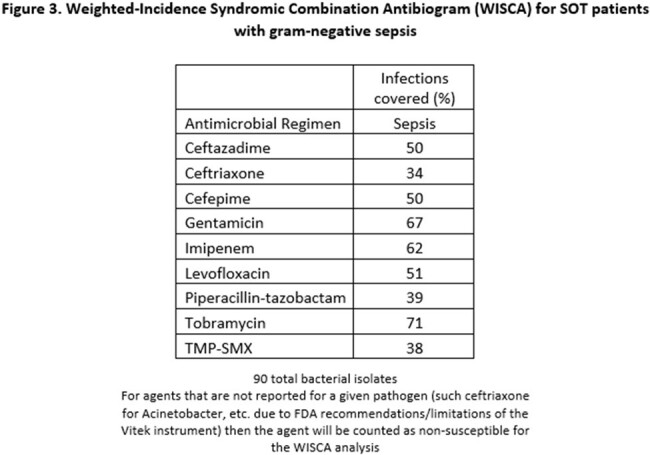

**Conclusion:**

Antimicrobial resistance rates were higher in SOT patients with Gram-negative sepsis compared to the non-SOT hospital population. Imipenem was a more active empiric Gram-negative antimicrobial for sepsis in the SOT patients compared to the institutional recommendation of piperacillin-tazobactam.

**Disclosures:**

**Scott Borgetti, MD**, GSK: Grant/Research Support **Alan E. Gross, PharmD**, Becton Dickinson Co: Advisor/Consultant

